# Nail Properties and Bone Health: A Review

**DOI:** 10.3390/jfb9020031

**Published:** 2018-04-23

**Authors:** Pouya Saeedi, Amin Shavandi, Kim Meredith-Jones

**Affiliations:** 1 Department of Human Nutrition, University of Otago, Dunedin 9054, New Zealand; pouya.saeedi@gmail.com; 2 Department of Food Science, University of Otago, Dunedin 9054, New Zealand; 3 Centre for Bioengineering & Nanomedicine, University of Otago, Dunedin 9054, New Zealand; 4 Department of Medicine, University of Otago, Dunedin 9054, New Zealand; kim.meredith-jones@otago.ac.nz

**Keywords:** nail plate protein, collagen, nail mineral composition, bone mineral density, Raman spectroscopy

## Abstract

Physicochemical properties of nail may offer valuable insight into the health of bone. Currently, dual-energy X-ray absorptiometry (DXA) is the gold standard technique for evaluating bone health through bone mineral density (BMD). However, only 70% of fractures are explained by low BMD according to DXA. Therefore, the World Health Organisation recommended the need for the development of alternative methods of assessing bone health. Keratin and collagen type I are major proteins in nail and bone, respectively. Both of these proteins undergo post-translational modifications, with a possible correlation between the degree of post-translational modifications in keratin and collagen. Raman spectroscopy is a technique used to detect changes in protein composition and structure. As changes in protein function and structure may be associated with the development of osteoporosis, Raman spectroscopy may be a valuable adjunct to assess bone health and fracture risk. This review critically evaluates various methods and techniques to identify the link between nail properties and bone health. The strengths and limitations of various studies and the potential use of nail protein and minerals to evaluate bone health have been also presented.

## 1. Introduction

Bone is a metabolically active tissue. Bone remodeling, i.e., bone formation and resorption, continues throughout life. The imbalance between this coupling process leads to excessive bone loss, little bone formation, or the combination of both. This phenomenon can cause osteoporosis or porous bone, which increases the risk of bone fracture. Calcium (Ca) and magnesium (Mg) are important components of human bones, and their deficiencies are associated with the development of osteoporosis [1,2]. Although medical interventions are indicated and proven to be efficacious with respect to fracture prevention [3], early diagnosis of osteoporosis is essential.

Bone densitometry is the gold standard for diagnosing osteoporosis and dual X-ray absorptiometry (DXA) is the most commonly used densitometry technique [4]. DXA technology also incorporates other measurements that have added value in fracture risk assessment, such as hip structural analysis and trabecular bone score [5]. In addition, DXA can detect skeletal sites that have low bone mineral density, but it is not able to identify the microstructure of bone, crystal shape and size, and the interconnectivity of bone porous structure. DXA is also not able to detect microfractures or identify individual bones at risk of fracture [6,7]. Furthermore, deficiency of the collagen protein in bone structure has been shown to be associated with bone fracture [8] and DXA is not able to assess the role of proteins in bone health. Therefore, alternative methods to detect bone architecture are required. 

Finger and/or toenails may be good indicators of metabolic changes occurring in the body, as they are in contact with periosteum of the phalangeal bone. Therefore, the physiological and pathological processes of blood and bone might influence the nail mineral content [9,10]. Thus, the mineral composition of the nail plates might be a suitable adjunct to bone densitometry to monitor bone health, i.e., the bone mineral metabolism pattern. Fingernail plates grow at a rate of 3.5 mm per month and different components including drugs, toxins, and biomarkers are passed into the nail tissue [9]. Nail samples are also easy to collect, transport, and store [11]. Therefore, nail clippings have been used for the detection of certain toxic components, exposure to heavy metals [12], nutritional imbalances such as iron deficiency [13], and to examine the relationship between the concentration of fingernail microelements with coronary heart disease and hypertension [14]. Studies have also evaluated the level of minerals such as zinc (Zn) [15] and selenium (Se) [16,17] in toenails and their possible association with the risk of myocardial infarction. 

**Figure 1:**
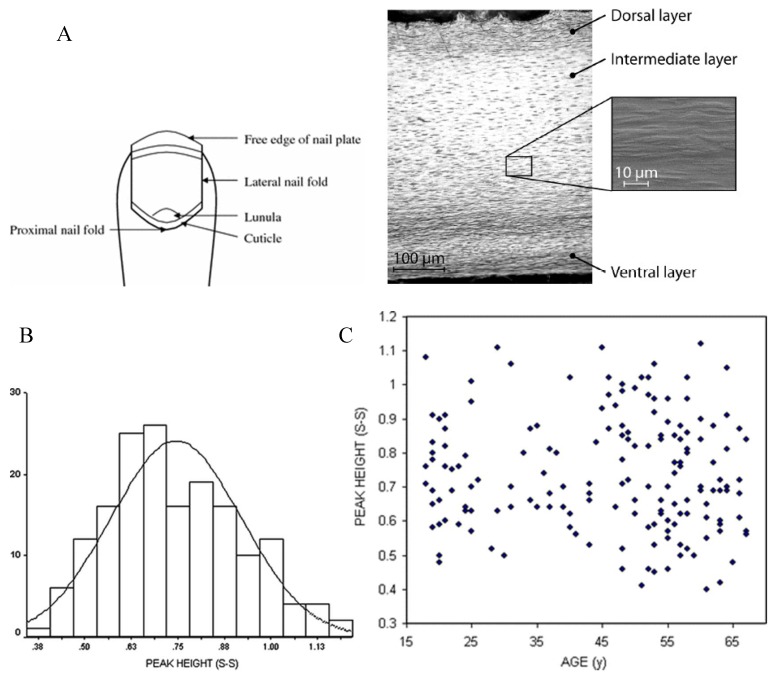
(**A**) Schematic and cross-section of the nail plate. The nail clipping is normally obtained from the nail plate edge [33]; (**B**) Histogram shows disulphide content of nails obtained from 169 women subjects; (**C**) Disulphide content of nails obtained from 169 women subjects in relation to age. The figures taken from [31], with permission of Springer.

In addition to the mineral content of nail, human nail plates also have a three-layered protein structure which includes alpha keratin, keratin microfibrils in the globular matrix, and keratin associated proteins (Figure 1A) [18]. Keratin is the main protein in nail, while collagen is the main protein in bone. Both keratin and collagen experience non-enzymatic and post-translational modifications that can be detected using techniques such as Raman spectroscopy. Post-translational changes that occur in nail disorders may also be associated with disorders in bone collagen (Table 1) [19,20]. Thus, nail mineral and protein content might be a useful alternative or complementary method for the screening and detection of bone metabolism disorders. This review summarises and critically evaluates studies that have examined the relationship between the physicochemical properties of human nail plates and bone health. 

## 2. Association between Nail Mineral Composition and BMD

Calcium (Ca) is the major elemental composition of bone and its deficiency is usually associated with osteoporosis [21]. Magnesium (Mg) also plays an important role in adjusting and controlling bone metabolism. The nail plate is in contact with the osteogenic layer of phalangeal bone for four to five months and is influenced by pathological and physiological changes in bone, such as changes in the content of Ca and Mg [10]. Nail also has deposits of Ca and Mg, which can be measured using techniques such as instrumental neutron activation analysis (INAA) [22]; a technique that is relatively fast and safe. 

Using INAA, the possible relationship between Ca and Mg content of toenail and BMD at the index finger from 220 women aged between 40 and 70 years was measured and shown to be very weak (correlation coefficients ranging from 0.03 to 0.18). The analysis was performed twice in a five-year interval and the authors reported a significant decrease in Ca density, but not Mg density. Given the weak correlation between the tested nail minerals and BMD, the authors suggested that the INAA method might not be a sensitive or reliable enough tool for osteoporosis screening [22]. However, in this study, nail mineral (Ca and Mg) content was compared to radiometrically measured BMD and not to bone mineral content. Thus, the weak correlation could be explained, because comparisons were made between two un-identical parameters of nail and bone. The difference could also be due to the site the nail was taken from (toenail) and the site BMD was tested at, i.e., the index finger. In addition, the radiometric method used to measure BMD in the index finger is considered to be insensitive, which may partly explain the insignificant relationship [10].

In a study by Bahreini et al. [23], laser breakdown spectroscopy (LBS) was used to measure the elemental composition of fingernails in 99 subjects (22–89 years), including 27 healthy, 47 osteopenic, and 25 osteoporotic adults. The association between fingernail minerals and BMD was investigated. Although the results demonstrated a relationship between nail mineral composition (Ca) and BMD, the validation results were not promising. In the validation sample, only 38.4% of the cross-validated cases were classified correctly. In addition, there was no correlation between the elemental composition of fingernails and risk of fracture or osteoporosis [23]. A study in older women (60–69 years) has demonstrated relationships between Ca and Mg and BMD [10], whereas others have not reported similar findings [11,24]. 

In a study in elderly women (≥80 years), a significant positive correlation was reported between the Ca to Zn ratio (Ca/Zn) in fingernails measured using atomic absorption spectroscopy (AAS) and BMD measured with DXA [25]. In addition, the Zn concentrations in fingernails were significantly but negatively correlated with BMD (r = −0.39). A negative relationship between Zn concentration and BMD has also been reported in Iranian osteoporotic women aged 36–60 years [24]. The contradictory findings between studies might reflect the differences in the health status and age of the sample populations, as well as the analysis techniques in these studies. 

Furthermore, higher levels of Zn and a significantly lower ratio of Ca/Zn and Mg/Zn were observed in the nail plates of osteogenesis imperfecta (OI) patients compared to normal controls. OI is a genetic metabolic disorder that affects the synthesis of collagen type Ι, where a minor trauma can result in fractures [26]. The high Zn content of fingernails in OI patients might be related to the disease process, which may increase Zn release from the bone [26]. Therefore, nail samples could potentially be used for the identification of OI patients. However, the pathomechanism of Zn release from bone and its accumulation in nails need to be revealed through further studies and for correctly diagnosing OI, the clinical assessment needs to be coupled with genetic mutation analysis [27].

The lack of consensus between studies may be related to the methodology used for measuring the mineral content and the population’s age or health status of those tested. In addition to the methods used to analyse nail samples, the variety of techniques used in the collection of nail samples may explain the discrepant results. Another methodological consideration is the location the nail sample is taken from. Nails on the thumb and little finger grow slower compared to other fingernails. Slower growing nails are in contact with the body for a longer time and therefore may have a different mineral content compared to other fingernails. Future studies should report the finger that has been used for collecting the nail plate samples. Identifying the finger used to supply the nail sample is not routinely reported in the literature, which might partly explain the variation in findings. 

## 3. Association between Nail Protein and BMD 

Keratin is the main protein constituent of human nail. The cysteine content of keratin determines disulphide (S–S) bonds and the physiochemical properties of nails. The formation of S–S bonds is essential for the protein’s structural integrity. A disorder in the metabolism of sulphur can negatively affect the S–S bonds and result in abnormalities in keratin containing tissues such as nail. The presence of S–S bonds from sulphur containing amino acids (e.g., cysteine and methionine) can be determined using a technique called Raman spectroscopy. In Raman spectroscopy, the molecules are vibrated after being subjected to a laser source. The recorded spectra contain information on the vibration of the molecules, which can be used to differentiate between various Raman active molecules [28,29]. Raman spectroscopy is non-destructive and does not require specific sample purification or extraction. It is also possible to acquire information about the secondary structure of proteins such as vibrations of amide I and III [30]. 

Given the similarities in structure and composition of nail keratin and bone collagen proteins, changes in the nail keratin properties may be a marker of osteoporosis [29]. As Raman spectroscopy can measure the degree of protein sulphating in fingernails, the technique may be used to evaluate bone health. In a patented application in the USA, Raman spectroscopy was used to demonstrate variation in conformations of the S–S bonds at 510 cm^−1^ and C–S bridges at 625 cm^−1^ and 641 cm^−1^, which were then associated with nail and bone health properties. The height of the S–S peak was also demonstrated to vary between subjects, with an approximately normal distribution (Figure 1B), and it was not associated with subjects’ age (Figure 1C) [31]. The patentee suggested that Raman spectroscopy of nails was a useful diagnostic tool to assess bone health [32].

**Figure 2:**
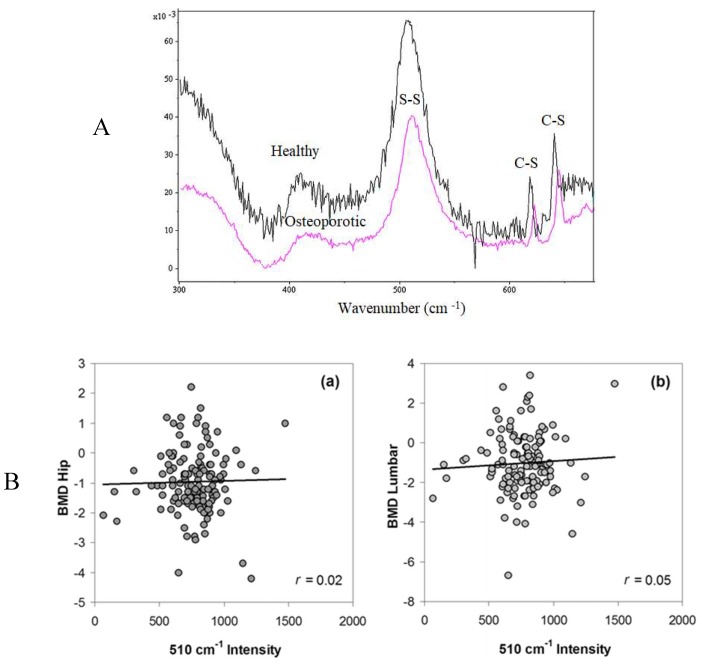

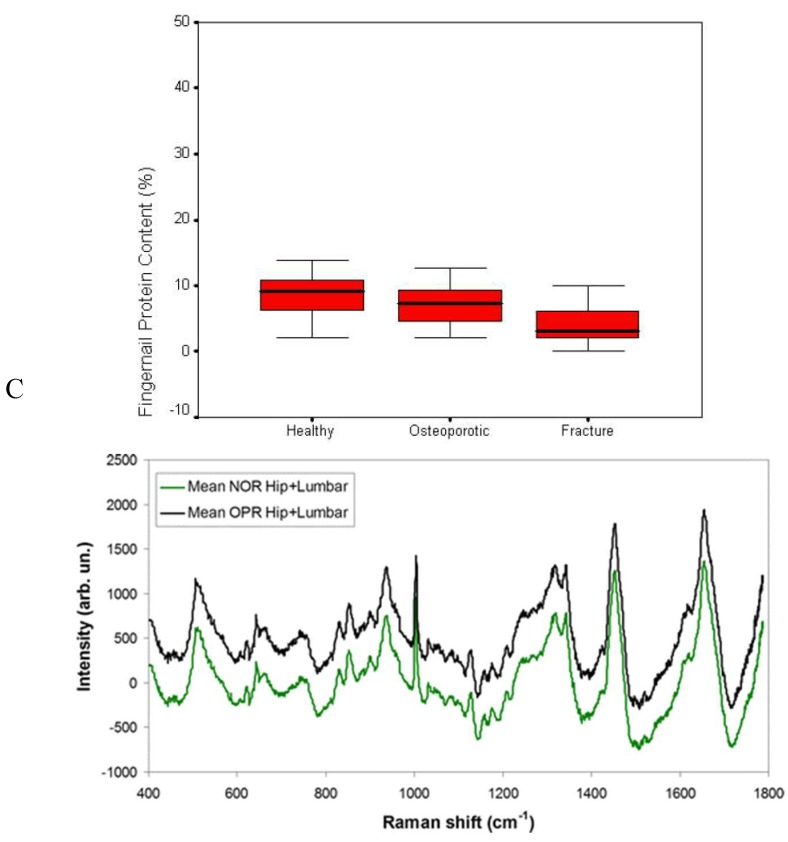
(**A**) Raman spectra from healthy (top) and osteoporotic subjects. The nail samples from osteoporotic patients had a lower S–S bond content than the healthy group; (**B**) Relationship between disulphide peak (∼510 cm^−1^) intensities and the BMD scores for the (**a**) hip and (**b**) lumbar spine groups. The nail Raman spectra arranged based on the osteoporosis presence in both hip and lumbar regions and compared to normal specimen. As shown, there were no relevant differences between these groups. The figures are taken from [33,35] with permissions from Springer. (**C**) The fingernail protein content in the patients with fracture was significantly lower than other tested groups. Comparison fingernail protein content in three study groups [42].

The same patent group [34] also evaluated the brittleness and S–S bond content of nails using nano indentation and Raman spectroscopy. The nail plate samples were collected from women with and without osteoporosis. The nails from patients with osteoporosis had a lower mean modulus compared with non-osteoporotic women. Additionally, lower S–S bonds were detected in the group with low BMD. The relationship between a lower content of S–S bonds and higher brittleness in the tested nails was not discussed. This group also investigated the effect of the reduced content of keratin S–S bonds on nail mechanical strength. The authors reported that the mechanical rigidity of nail is associated with the nail protein disulphide content and the fingernails of women with a history of fracture showed a lower disulphide content than women with no history of fracture (Figure 2A). 

The peak of the S–S bonds may also be related to bone health. More than 7% of the total amino acid content of nail is cysteine, which has a high content of sulphur. Raman spectra analyses of the nail sulphur bondings are in the region of 300–700 cm^−1^. In particular, the peak at 510 cm^−1^ indicates the S–S bond. Higher peak intensities at 510 cm^−1^ have been reported for healthy nails compared to osteoporotic patients, indicating more cysteine and S–S bonds. In addition to a greater number of S–S bonds, a C–S spectral shift has been reported in the nails of adults with osteoporosis, which may be related to the S–S bond cross linking density [29,31]. Other studies [33] have also reported a 25% reduction in nail keratin in osteoporotic women and peaks of S–S bonds have been demonstrated to be sharper in nails from healthy adults compared to nails from adults with osteoporosis, indicating the higher content of S–S bonds in healthy nails. All of these studies were patented studies, and mainly cross-sectional with small sample sizes, which may have precluded detecting changes in the protein composition of the nail. However, Mussatto et al. [35] have not demonstrated a relationship between the keratin properties of the nail and bone health (Figure 2B). The discrepant results may be due to the lack of a proper spectra baseline correction model. Positive relationships have been demonstrated by Beattie et al. [36], who developed a baseline correction model using a singular value decomposition (SVD)-based method.

Using a baseline spectra correction model, Beattie and associates evaluated the amino acid profile of nail keratin samples from both fracture and non-fracture samples [36]. The keratin from the fracture group had no evidence of S–S bonds and had a low content of basic and hydroxyl amino acids compared to the control samples. The fractured samples also had a higher content of aromatic amino acids. In addition to the observed difference between individual amino acids, the alpha helices and beta sheets were more ordered and structured in the non-fracture samples compared to the fractured group. This change in the protein structure could be related to the reduction in the content of S–S bonds. A lower number of S–S bonds indicates less cross-linking between the chain of the keratin, which leads to a keratin chain with a lower integrity and structure.

Most of the studies on protein properties of the fingernail and its association with bone health have not considered the relationship between parameters such as the width, area, or intensity of S–S bonds with bone densitometry data. To-date, one cross sectional study by Cummins et al. [37] has analysed the width of S–S bonds and found no relationship between Raman spectra data and BMD.

In people with osteoporosis, the activity and number of osteoblasts decrease and the function and activity of osteoclasts increase. When the bone loses its mineral content, collagenase begins to reabsorb collagen. Under disease conditions such as osteoporosis, the reduction in the density of bone may indicate a deficiency in the formation of a protein matrix. Therefore, the reduction in the amount of Ca may reduce the quantity of collagen and S–S bonds in the bone [38]. To assess this, nail Raman spectroscopy was carried out in a sample of 213 women (61.5 ± 9.7 years) [35]. The nail spectra and the chemical composition of participants with and without osteoporosis were similar and no differences were seen between the disulphide peaks. There was also no association between BMD values and the Raman spectra intensity of the nails at 510 cm^−1^ or S–S bonds. 

Raman spectroscopy may also be a valuable method to identify those at risk of fracture. Two recent studies [36,39] have collected nail samples from 633 postmenopausal British and Irish women and analysed them using Raman spectroscopy. Forty-two percent of the tested population experienced a fracture due to fragility. Comparing DXA measured BMD, Raman spectroscopy, and biomarkers of bone health data, the authors reported Raman spectroscopy as the most accurate method to differentiate between individuals with and without fracture (*p* = 0.003). As inter and intra S–S bondings are required for the synthesising, folding, and stability of collagen, the observed changes in Raman spectroscopy of nail S–S bonds may indicate changes and disorders in bone. However, as this was a cross-sectional study in patients with existing fracture, the Raman spectroscopy studies of this kind may not be able to predict risk of future fracture. Furthermore, the absence of fracture may simply indicate a lack of low impact trauma. In addition, the group with no fracture may not have been homogeneous in terms of bone health and therefore the results of these studies do not provide strong evidence that Raman spectroscopy can be used as a diagnostic tool or to predict fracture risk. 

## 4. Association between Nail and Bone Proteins

Bone proteins such as osteonectin and signaling proteins of growth factor-B (TGF-B) contain cysteine and S–S bonds. Osteonectin is vital for bone structure, remodeling, and maintenance [40]. Osteonectin and TGF-B-signalling proteins are also composed of S–S bond dimers made of two chains of polypeptide that are highly rich in cysteine residues [40]. Cysteine residues and S–S bonds are important for the three-dimensional structure of the bone morphogenic protein 2 (BMP-2), a subset of the TGF-B signalling proteins superfamily [41]. Human bones are made up of either cortical bone or trabecular bone, and trabecular bone is known to decrease in mineral with age. Given that osteonectin content is 20–40 folds higher in human trabecular bone compared to cortical bone, a nail S–S bond measurement may be a viable method to measure the health of trabecular bone. 

Other bone formation markers including osteocalcin, serum C-terminal telopeptides of type I collagen (serum cross laps), serum osteoprotegerin, and serum sRANKL have been measured and compared to nail protein in a large sample of postmenopausal women [42]. Nail protein content was significantly correlated with the concentration of serum cross laps [42] after controlling for BMD, age, and body mass index (BMI). The nail protein content in patients with fracture was also significantly lower than both healthy and osteoporotic adults (Figure 2C). Nevertheless, it was not clear how many of the patients exhibited fracture and some of the individuals with fracture history had zero or negative values for the fingernail protein content. 

### Nail Viscoelastic Properties and Bone Health

It has been suggested that the viscoelastic properties of nail may be associated with bone health, as load and time displacement tests have shown different depths of indentation and higher creep in nails of osteoporotic adults [29]. To measure the depth of indentation and creep, a maximum load of 2 mN was applied to the nail samples and held for 2000s, followed by unloading. This loading phase resulted in a viscoelastoplastic response. The nail samples reacted on this load with a deformation (i.e., creep). While the elastic-plastic loading segment of nails was identical for both healthy and osteoporotic individuals, the nails from osteoporotic adults showed higher displacement over the tested time. The conclusion was that nails of adults with osteoporosis showed differences in the time-dependent response to mechanical load, but not in the hardness nor the elastic modulus. Moran et al. [33] and Pillay et al. [34] evaluated the elastic modulus of fingernail plates and did not report a significant association between the nail S–S bond content and the nail’s modulus. The lack of finding may have been because the authors tested the polymeric elastic region of the nail plates, which has both a viscoelastic and viscoplastic property, but it is the visco-elastoplastic property that is mostly affected by the density of the S–S bond and not the hardness or elastic modulus properties.

## 5. Conclusions 

Nail samples can be used to measure nail properties such as hardness, elastic modulus, and content of the S–S bonds. Nail plate sample collection is relatively easy, fast, and inexpensive. Methods to determine bone health using nails show promise, but studies investigating nail mineral and bone density are hampered by the methodology used to measure these parameters. Methods such as Raman spectroscopy have been able to detect differences between fracture and fracture-free groups. However, variations in physicochemical properties of nail and differences in nail growth speed in different individuals limit measuring bone mineral content through the nail and consequently evaluating bone health through the nail. Furthermore, studies have not been consistent and the measurement of nail plate protein and minerals cannot yet be considered as a routine practice for the screening of bone disorders. Given that Raman spectroscopy can provide insight into bone architecture and not just bone mineral density, it may be a promising investigational tool for the measurement of overall bone health and fracture risk. However, it is hardly plausible in clinical practice that Raman spectroscopy will be an alternative to DXA in the clinical assessment of bone health. Future studies should investigate the use of Raman spectroscopy to directly measure the protein properties of bone through the skin at various body sites.

## Figures and Tables

**Table 1:** A summary of studies on the possible relationship of the human nail properties and bone health status.

Sample Population	Country	Measurements	Result	Ref
*n* = 135, 61 osteogenesis imperfecta (OI) males and 74 females 1–61 years old. 61 males and 74 females healthy controls	Japan	AAS	Significantly higher Levels of Zn in OI nails. The ratios of Ca/Zn and Mg/Zn in OI nails differed significantly from those in controls. Zn Levels in fingernails may reflect abnormal Zn metabolism in OI.	[26]
*n* = 123, Postmenopausal women 53–56 years old	Iran	DXA, X-ray Radiography Serum Osteocalcin and Cross laps concentrations	Fingernail protein content significantly correlated with serum. Cross laps concentration, lumbar spine BMD, and total hip BMD fingernail protein content predicted vertebral fracture.	[42]
*n* = 159, ≥65 years old	Turkey	DXA, AAS	No statistically significant difference in nail Ca levels between osteoporotic and non-osteoporotic patients and similarly between vitamin D deficient and normal patients. No change in nail Ca concentrations in osteoporosis and vitamin D deficiency.	[11]
*n* = 2, One healthy 38 years old male; One osteoporotic 65 years old female	USA	Nano indentation Raman spectroscopy	Osteoporotic nail samples showed greater creep than non-osteoporotic nail samples.	[29]
*n* = 169, Females aged 18–67 years old	Ireland	Raman spectroscopy DXA	Lower disulphide content in women postmenopause. The disulphide content of nails from women with a history of fracture was significantly lower than those with no history of fracture.	[31]
*n* = 22, 9 osteoporotic 13 non osteoporotic	Ireland	Nano indentation Raman spectroscopy	Significantly lower S–S bond content fingernail in the osteoporotic group. The differences in nano indentation were not statistically significant.	[33]
*n* = 22, 9 osteoporotic 13 non osteoporotic	Ireland	Nano indentation Raman spectroscopy	The differences in nano indentation were not statistically significant. Sharper disulfide bond peak for nail from control group than for the nail from the osteoporosis group	[34]
*n* = 184, 69 women 115 men and 20–80 years old	Japan	AAS for Ca and Mg DXA	Mineral content may be utilized as one of the indicators of bone mineral metabolism. Fingernail and toenail Ca concentrations decreased with age in both men and women. Postmenopausal women had lower nail Ca concentrations than premenopausal women. LBMD showed a significant positive correlation with nail Ca content.	[10]
*n* = 8, Postmenopausal women 36–60 years old	Iran	ICP-AES for Zn, Copper and Mg and DXA	No correlation between case and control groups in trace minerals in the nail samples.	[24]
*n* = 99, 27 healthy, 47 osteopenic, and 25 osteoporotic	Iran	LIBS Sodium, potassium, Ca and iron, Mg and silicon	The classification between nail samples of healthy, osteopenic, and osteoporotic subjects is attainable. Some evidence for association between osteoporosis and elemental composition of fingernails measured by LIBS.	[23]
*n* = 633, Postmenopausal women	UK/Ireland	Raman spectroscopy and DXA	Raman spectroscopy can provide insight into a subject’s fracture risk. Increasing disorder in S–S bonding orientation transition from alpha helical secondary structure to random differences in the amino acid composition of the two groups.	[36,39]
*n* = 220, Women 54.6 ± 9.1 years old	Netherland	INAA for Ca and Mg DXA	Ca and Mg measurements in nail clippings by INAA cannot be used for screening purposes in the prevention of osteoporosis.	[22]
*n* = 159, Women 18–67 years old	Ireland	Raman spectroscopy DXA	Raman spectroscopy of keratin may have potential as a diagnostic tool for screening bone quality in large populations.	[37]
*n* = 213 women	Brazil	Raman spectroscopy DXA	No differences in the mean Raman spectra of nails of groups with and without osteoporosis. BMD and fracture risk could not be assessed by the nail keratin features.	[35]

Atomic absorption spectrophotometer (AAS). Instrumental neutron activation analysis (INAA). Laser-induced breakdown spectroscopy (LIBS). Dual X-ray Absorption (DEXA). Inductively Coupled Plasma-Atomic Emission Spectrometry (ICP-AES).
